# Iranian international headache congress, 2015

**Published:** 2016-01-05

**Authors:** Mansoureh Togha

**Affiliations:** ^1^ Professor, Iranian Center of Neurological Research, Sina Hospital, Tehran University of Medical Sciences, Tehran, Iran

**Keywords:** Congresses, Headache, Migraine, Iran

The 4^th^ Iranian International Headache Congress was held in Tehran, Iran, from September 16 to 18, 2015. The congress was supported by Iranian Neurological Association under the auspicious of International Headache Society. 340 physicians and researchers participated in the congress among them Professor A. Rapoport, Professor R. Cowan, Professor H. Bolay, Dr. F. Maniyar, and Dr. H. Ansari were the international guests. 

The congress was managed by Professor M. Togha and nicely supported by the president of the Neurological Association, Professor H. Pakdaman. The main aim of the congress was to familiarize neurologists and other specialists with the latest scientific achievements in the field of “Headache”.

The interesting characteristic of this congress was that abstracts and most of the full texts of lectures were available online on our website from the first day of the congress. In addition, the hard copies of the abstract were offered to the participants on registration. 

Accordingly, several scientific programs were held during 3 days. The programs were divided into the following five categories:

***1. Main Lectures***

Totally, 35 lectures were presented by headache specialists and physicians who were considered expert in a specific field related to primary or secondary headaches.

Accordingly, we can refer to the basic lectures, like pathophysiology of migraine [CGRP (calcitonin gene-related peptide)] presented by Professor A. Rapoport, and basic science (how we could use in animal model) presented by Professor H. Bolay. Besides, different speeches on updates in the field of diagnosis and treatment of primary headaches were given, like non-cephalic migraine equivalents by Professor R. Cowan, optimizing treatment of acute migraine attack by Dr. F. Maniyar, and cluster headache by Dr. H. Ansari. 

Different speeches were offered on migraine variants, secondary headaches and related subjects such as metabolic headache by Professor M. Ghafarpoor; ictal epileptic headache by Professor M. Motamedi; migraine and vertigo by Professor M. Togha, and drug interactions in headache treatment by Dr. L. Kuti.

***2. Case Presentation***

This part was one of the popular sections of the congress moderated by Dr. M. Nabavi, and five challenging cases were discussed in an interactive presentation.

***3. Workshops***

In this section, two workshops were provided, in the first of which mainly younger neurologists were trained on techniques of botulinum toxin injection for chronic migraine; and the techniques of nerve blocks in headaches were touched in the second workshop. These workshops were nicely moderated by Dr. H. Ansari. 

***4. Panels***

Two panels were held during the congress. The first panel discussed different aspects of idiopathic intracranial hypertension and idiopathic intracranial hypotension, and the other one reviewed different unapproved but effective treatment modalities on headaches including migraine surgery, nutritional aspects on headache, the effect of exercise on headaches, and the suggestions of Iranian traditional medicine for headaches.

***5. Residents Scientific Competition***

This was nicely conducted by Dr. A. Okhovat with cooperation of Dr. S. Ahmadi-Karvrigh and Dr. E. Hesami. In this part, the competition was between the selected neurology residents from 13 universities of different cities of Iran in the primary and final sections and the first, second and third winners were received valuable presents. 

The valuable assistance of Dr. S. Baghizadeh and Mr. H. Ghasemi and nice cooperation of Dr. H. Paknejad, Dr. A. Okhovat, Dr. S. Haghighi, Dr. N. Yamani, Dr. F. Vahabi, Dr. Advani, Dr. M. Ghafarpoor, A. Naser Moghadasi, S. Razeghi Jahromi and S. Habibi Moeini as well as, the other members of scientific and organizing committee had an important role in the better management of the congress.

The headache congress could unquestionably play an important role in promoting the status of knowledge in the field of headache in Iran. The positive feedback from the attendees was a good sign of achievement to the goal of the congress that was the presentation of high-quality programs meet their expectations. 

Indeed, attendance of distinguished international headache specialist with the cooperation of International Headache Society, and Iranian esteemed neurologists as lecturers and the active participants from different parts of Iran in response to invitation from Iranian Headache Association contributed to establish a good relationship between the Iranian Headache Association and International Headache Society.

**From left to right in the front:**
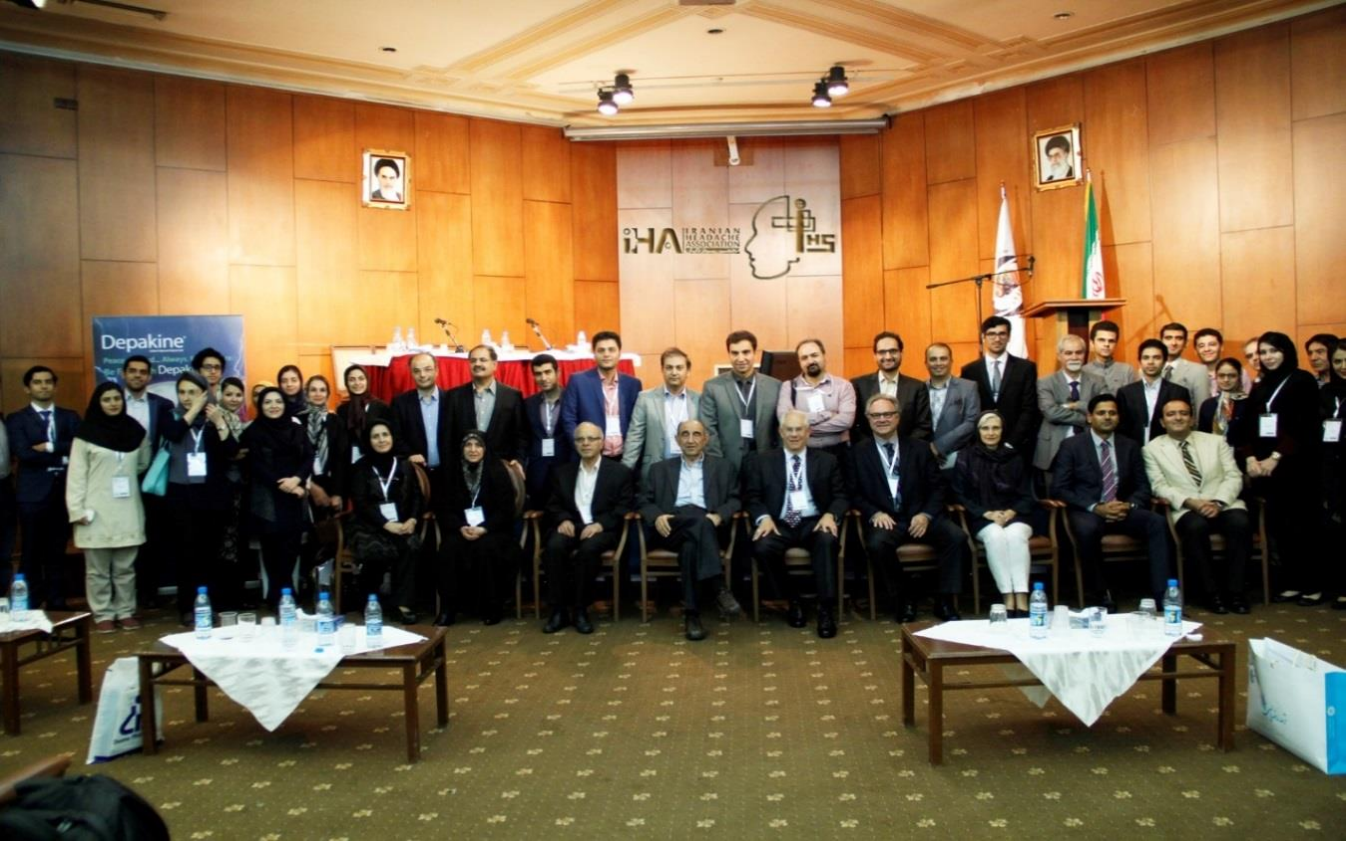
Dr. N. Beladimoghadam, Professor M. Togha, Dr. J. Adibeig, Professor H. Pakdaman, Professor A. Rapoport, Professor R. Cowan, Professor H. Bolay, Dr. F. Maniyar, and Dr. H. Ansari

## Notes:

**How to cite this article:** Togha M. **Iranian International Headache Congress, 2015**. Iran J Neurol 2016; 15(1): ???-60-1.

